# Probiotic Supplementation and its Potential Benefits in Type 2 Diabetes: A Meta-analysis

**DOI:** 10.2174/0118715303425888251212053249

**Published:** 2026-04-29

**Authors:** Marica Colella, Manuela Mandorino, Stefania Garzone, Maria Elena Maggiore, Loredana Capozzi, Massimo Bilancia

**Affiliations:** 1 Interdisciplinary Department of Medicine, Section of Microbiology and Virology, School of Medicine, University of Bari, 70124 Bari, Italy;; 2 Department of Theoretical and Applied Sciences (DiSTA), eCampus University, 22060 Novedrate, Italy;; 3 Istituto Zooprofilattico Sperimentale della Puglia e della Basilicata, 71121 Foggia, Italy;; 4 Unit of Statistics, Department of Precision and Regenerative Medicine and Jonian Area (DiMePRe-J), University of Bari Aldo Moro, 70124 Bari, Italy

**Keywords:** Probiotics, microbiota, type 2 diabetes, blood sugar, HbA1c, insulin, meta-analysis

## Abstract

**Introduction:**

This meta-analysis aims to evaluate the efficacy and safety of probiotic supplementation as an adjunct to standard therapy in patients with type 2 diabetes (T2D), with a focus on its effects on plasma glucose, HbA1c, and insulin levels.

**Methods:**

Specific keywords such as “Type 2 Diabetes”, “Probiotics”, and “Blood Sugar” were employed to identify relevant clinical and randomized controlled trials. A comprehensive search of PubMed, Scopus, and Web of Science, conducted between September 2010 and October 2024, retrieved 48 studies, of which only 9 met the predefined inclusion criteria. Studies were excluded if they did not involve patients with T2D, failed to specify the probiotic strains used, lacked a placebo-controlled arm, were not written in English, were not freely accessible, or did not report outcomes of interest.

**Results:**

Three separate meta-analyses were conducted to evaluate the primary and secondary outcomes of interest: plasma glucose, HbA1c, and insulin concentrations. Each analysis revealed statistically significant effects. The magnitude of the effect of probiotic supplementation in patients with T2D is expressed by a promising reduction in fasting plasma glucose levels (MD = -17.68; 95% CI: -21.72 to -13.63; *p* < 0.0001; I ^2^ = 0%; *p* for heterogeneity = 0.76); HbA1c (MD = -0.42; 95% CI: -0.67 to -0.17; *p* = 0.0009; I^2^ = 0.5%; *p* for heterogeneity = 0.40) and insulin (MD = -2.26; 95% CI: -2.87 to -1.65; *p* < 0.0001; I^2^ = 0%; *p* for heterogeneity = 0.67).

**Discussion and Conclusion:**

This meta-analysis included a total of 649 patients with T2D, who showed a reduction in levels of some clinical indicators at the end of the intervention period. These findings provide evidence supporting the use of probiotic supplementation as an adjunct to standard therapy for the management of this condition.

## 1 INTRODUCTION

More than 480 million people worldwide are currently affected by diabetes, and this number is expected to rise substantially in the coming decades (World Health Organization, 2016). Projections estimate that over 630 million individuals could be living with the condition by 2045 (International Diabetes Federation, 2017). The global healthcare costs associated with diabetes are estimated at approximately $827 billion, underscoring the immense and growing economic burden posed by this disease [1, 2].

The American Diabetes Association (ADA) defines diabetes mellitus as a group of metabolic disorders affecting carbohydrate metabolism. Diabetes can be classified into several clinical types, including type 1 diabetes, type 2 diabetes, gestational diabetes mellitus, and other forms resulting from various etiologies, such as exocrine pancreatic disorders, genetic factors, or drug-induced causes.

Diagnosis should be confirmed in patients exhibiting classic signs and symptoms, including excessive thirst and/or hunger, fatigue, frequent urination, weight changes, blurred vision, tingling or numbness in the hands or feet, recurrent infections, slow-healing wounds, or manifestations of diabetic complications such as retinopathy, nephropathy, neuropathy, impotence, or acanthosis nigricans [3].

Several diagnostic tests are available for type 2 diabetes; however, current guidelines prioritize methods that combine accuracy with ease of use, as summarized in Table **1** [4].

Type 2 diabetes (T2D) represents approximately 90% of all diabetes cases. It is influenced by environmental factors and host physiology, potentially leading to reduced life expectancy and severe, costly complications. Currently, no definitive cure exists for T2D, and management typically involves long-term use of antidiabetic medications. Therefore, exploring alternative therapeutic strategies to mitigate or reverse the progression of T2D remains a critical priority [5-8].

T2D is frequently associated with hypertension, oxidative stress, and dysregulated lipid metabolism. These factors, combined with inadequate diabetes management, contribute to both microvascular and macrovascular complications. Microvascular complications, which affect small blood vessels, include diabetic retinopathy, nephropathy, and neuropathy. Macrovascular complications involve larger blood vessels and encompass cerebrovascular disease, peripheral vascular disease, and coronary artery disease. Less common complications include stroke, weight gain, congestive heart failure, electrolyte imbalances, and peripheral vascular conditions such as diabetic foot [9].

Although T2D is most prevalent among elderly individuals, its incidence is increasing in younger populations, including children, adolescents, and young adults. This earlier onset is attributed to various causative and triggering factors, with obesity being the most prominent. Additional risk factors implicated in the pathogenesis of T2D include physical inactivity, excessive caloric intake, reduced total energy expenditure, genetic predisposition, low socioeconomic status, lower education levels, and alterations in gut microbiota [10].

The composition of the gut microbiota is closely associated with overall health. An imbalance in this composition, referred to as dysbiosis, has been linked to various pathologies through the promotion of a heightened inflammatory state [11].

Notably, patients with T2D have been reported to exhibit several microbiota-related alterations, including dysbiosis, increased abundance of butyrate-producing bacteria, enhanced intestinal permeability, elevated expression of microbial genes associated with oxidative stress, reduced expression of genes involved in the synthesis of specific vitamins, and higher concentrations of lipopolysaccharide (LPS) [12, 13].

Several studies have reported alterations in the gut microbiota of diabetic patients, characterized by a decrease in *Firmicutes* and an increase in *Bacteroidota*, *Proteobacteria*, and potential pathogenic opportunists, including *Escherichia coli*, *Enterocloster clostridioformis* (formerly *Clostridium clostridioforme*), *Hungatella hathewayi* (formerly *Clostridium hathewayi*), *Clostridium symbiosum*, *Bacteroides caccae*, *Thomasclavelia ramosa* (formerly *Clostridium ramosum*), and *Eggerthella spp*.

In this context, several studies have proposed probiotics as a supportive intervention for metabolic diseases, including T2D. Probiotics modulate the altered gut microbiota and can reduce inflammation, resulting in beneficial effects in patients with these conditions. Specifically, probiotic supplementation in T2D has been shown to improve peripheral insulin sensitivity, preserve intestinal barrier integrity, reduce systemic levels of lipopolysaccharide (LPS), and alleviate endoplasmic reticulum stress. [14].

Probiotic supplementation may represent a promising therapeutic approach for T2D and related metabolic disorders. Previous studies have demonstrated that several probiotic strains, including *Lacticaseibacillus rhamnosus*, *Akkermansia muciniphila*, and members of the *Bifidobacteriaceae* family, confer beneficial effects on metabolic health. For instance, supplementation with *Limosilactobacillus reuteri* ADR-1 or ADR-3 has been shown to reduce glycated hemoglobin (HbA1c) and serum cholesterol levels in patients with T2D [15]. Furthermore, a negative correlation has been observed between *Akkermansia muciniphila* and *Bifidobacteriaceae* abundance and markers of inflammation, insulin resistance, and T2D. These microorganisms produce short-chain fatty acids (SCFAs) and contribute to intestinal barrier integrity by reducing the translocation of lipopolysaccharides (LPS) into systemic circulation [16, 17]. Additionally, several studies have demonstrated mutualistic interactions between different intestinal microbiota strains, such as *Lacticaseibacillus rhamnosus* GG and butyrate-producing bacteria, as well as specific strains of *Bifidobacterium* (*e.g.*, Ba8145) and *Akkermansia muciniphila* [16, 18].

Although exogenous probiotics are generally beneficial to human health, they may, in some cases, affect the intestinal environment by competing with endogenous gut microbiota [19]. Bacterial infections and antimicrobial resistance represent critical safety concerns associated with probiotic use. Studies have indicated that supplementation with *Lactobacillus*, particularly *Lacticaseibacillus rhamnosus*, may induce bacterial translocation into the bloodstream, potentially resulting in bacteremia [20, 21]. Moreover, members of the *Bifidobacteriaceae* and *Lactobacillaceae* families have the potential to transfer antibiotic resistance genes to intestinal commensal pathogens [22]. Therefore, it is essential to carefully evaluate the risk-benefit ratio prior to recommending probiotics as adjunctive therapy for patients with diabetes and associated conditions [23].

The objective of this meta-analysis was to systematically review and compare all available clinical and randomized controlled trials assessing the efficacy of various probiotics as adjuncts to pharmacological treatment in T2D. Specifically, we evaluated changes in fasting plasma glucose, HbA1c, and insulin concentrations.

## 2 MATERIALS AND METHODS

This meta-analysis was conducted in accordance with the guidelines of the Cochrane Collaboration and the Preferred Reporting Items for Systematic Reviews and Meta-Analyses (PRISMA 2020), and it has been registered in the PROSPERO database https://www.crd.york.ac.uk/PROSPERO/view/CRD420251116306.)

### 2.1 Study Eligibility

We included all studies meeting the following inclusion criteria:

1. Randomized controlled trials or clinical trials
2. Comparison between probiotics and a placebo
3. Patients diagnosed with T2D undergoing pharmacological treatment
4. Studies reporting at least one of the clinical outcomes of interest
5. Free full-text articles
6. Articles published in English

Conversely, we excluded studies that met any of the following exclusion criteria:

1. Studies without a placebo group or where the “placebo group” was administered probiotics.
2. Studies in which the probiotic microorganism was not specified.
3. Studies with more than two intervention arms.
4. Studies involving patients with prediabetes or other forms of diabetes.
5. Studies that were not freely accessible.
6. Studies reported only as abstracts or conference presentations.
7. Studies that did not report any outcomes of interest.

Inclusion criterion number 6 and exclusion criterion number 5 could generate bias in the selection of articles for study.

### 2.2 Search Strategy and Data Extraction

The literature search was performed on PubMed, Scopus, and Web of Science, covering the period from September 2010 to October 2024. The keywords used were “Type 2 diabetes mellitus” AND “Probiotics” AND “Blood sugar,” with filters applied for “Humans”, “Clinical trials”, and “Randomized controlled trials.” This search retrieved 48 results, of which only 9 studies met the inclusion criteria and were included in the meta-analysis.

### 2.3 Endpoint

The primary objective of this study was to evaluate the effect of probiotic supplementation on plasma glucose concentrations, compared to baseline levels, in patients with T2D. Secondary outcomes included the mean changes in HbA1c percentage and insulin concentration following probiotic administration. Patients who experienced adverse reactions to probiotics were excluded from all studies included in this meta-analysis.

### 2.4 Statistical Methods

The overall mean difference (MD) effect was estimated using both fixed-effects and random-effects models, with results presented in a forest plot [24] Heterogeneity was assessed using the I^2^ statistic, which quantifies the proportion of variability attributable to heterogeneity rather than to sampling error [25]. Publication bias was assessed using funnel plots [26]. In all three studies considered, the effect size was expressed as the Standardized Mean Difference (SMD), corrected to Hedges’ *g*, thereby allowing for the combination of studies measuring the same outcome but employing different scales or units. For the random-effects model, the between-study variance t^2^ was estimated using the DerSimonian–Laird method, while the corresponding p-value was derived from Cochran’s Q-test under the null hypothesis that all studies share a common true effect size. All analyses were conducted in R version 4.4.2, using the Meta package (version 8.0-1) [27, 28].

## 3 RESULTS

As illustrated in Fig. (**1**), the search strategy identified 48 studies; however, applying our eligibility criteria resulted in the inclusion of only 9 studies, which were fully analyzed.

### 3.1 Study Selection and Characteristics

The total population across the nine clinical trials comprised 649 patients with T2D: 328 in the experimental (probiotic) group and 321 in the control (placebo) group. Patient characteristics were comparable between groups, with ages ranging from 18 to 75 years. In all included trials, the following parameters were evaluated in both arms: fasting plasma glucose (FPG, mg/dL), HbA1c (%), and insulin concentration (μIU/mL). Among the selected studies, all assessed FPG, seven evaluated insulin levels, and six measured HbA1c [29].

The duration of treatment applied in the nine studies investigated has an average value of 11.2 weeks and a median of 12 weeks. Respectively, the studies that provided for probiotic supplementation of longer and shorter duration are those by Zikou *et al*. 2023 (24 weeks) and Asemi *et al*. 2015 (6 weeks).

As regards the bacterial species of probiotic strains administered to treated patients, the most represented were *Lactobacillus acidophilus* , *Lacticaseibacillus casei* , and *Bifidobacterium bifidum* (administered to patients in 7, 5, and 4 studies, respectively); *Bifidobacterium longum* and *Streptococcus salivarius* ssp. *thermophilus* were used in 3 studies; *Lactiplantibacillus plantarum* , *Lactobacillus delbrueckii* ssp. *bulgaricus,* *Bifidobacterium breve* , and *Bacillus coagulans* were used in 2 studies each. Only one of the nine selected studies administered the following species: *Lactobacillus delbrueckii* subsp. *lactis, Lactobacillus helveticus, Lacticaseibacillus rhamnosus, Bifidobacterium longum* subsp. *infantis, Bifidobacterium animalis* subsp. *lactis, Saccharomyces boulardii, Propionibacterium freudenreichii* ssp. *schermanii* , *Propionibacterium acidipropionici* , *Acetobacter aceti* , *Ligilactobacillus salivarius* , and *Lactococcus lactis* ssp. L *actis* .

This analysis did not allow for an evaluation of the safety of using these bacterial strains. Indeed, many of the studies analyzed did not mention any adverse effects recorded in patients.

### 3.2 Fasting Plasma Glucose

The mean difference between the experimental and control groups (common effect model: MD = -17.68; 95% CI: -21.72 to -13.63; *p* < 0.0001; I^2^ = 0%; p for heterogeneity = 0.76; Fig. **2**) indicates a significant reduction in fasting plasma glucose (FPG) levels in the experimental group following probiotic supplementation. No heterogeneity was observed among the studies, as reflected by the I^2^ value of 0.0% (95% CI: 0.0% to 64.8%). Additionally, a funnel plot analysis of these nine studies (Fig. **3** - Left) showed an overall symmetrical distribution, suggesting no significant publication bias (Fig. **3** - Right). However, several studies exhibited high standard errors, including those by Tajabadi-Ebrahimi *et al*. (2016), Khalili *et al*. (2018), Jiang *et al*. (2024), and Savytska *et al*. (2024) [30-33]. A contour-enhanced funnel plot (Fig. **3** - Left) was generated to clarify statistical significance. Among the studies, three demonstrated a significant effect size (*p* < 0.01): Asemi *et al*. (2013), Zikou *et al*. (2023), and Firouzi *et al*. (2016) [34-36].

### 3.3 HbA1c

At the end of the treatment, the probiotic groups showed a significantly greater reduction in mean difference compared to the placebo group (common effect model: MD = -0.42; 95% CI: -0.67 to -0.17; *p* = 0.0009; I^2^ = 0.5%; *p* for heterogeneity = 0.40; Fig. **4**). This analysis included data from five studies, as not all nine trials reported this parameter. The funnel plot (Fig. **5** - Left) demonstrated an overall symmetrical distribution. A very low degree of heterogeneity was observed (*I*^2^ = 0.5%; 95% CI: 0.0% to 79.3%) [32-35, 37].

However, the data also exhibited an asymmetrical pattern, which prompted the generation of a contour-enhanced funnel plot (Fig. **5** - Right). This analysis identified that the studies showing a significant effect (*p* < 0.01) were those by Madempudi *et al*. (2019) and Jiang *et al*. (2021) [32, 34, 35, 37, 38].

### 3.4 Insulin

The mean difference indicates a significant effect between groups at the end of the study (common effect model: MD = -2.26; 95% CI: -2.87 to -1.65; *p* < 0.0001; I^2^ = 0%; p for heterogeneity = 0.67; Fig. **6**). This analysis included data from six studies. The funnel plot (Fig. **7** - Left) shows that all studies fall within the expected funnel shape, suggesting the absence of publication bias. A low degree of heterogeneity was observed (I^2^ = 0.0%; 95% CI: 0.0% to 74.6%). We also generated a contour-enhanced funnel plot (Fig. **7** - Right), which revealed that only two studies (Asemi *et al*., 2015, and Firouzi *et al*., 2016) presented a *p*-value lower than 0.01 (*p* < 0.01) [34, 36].

## 4 DISCUSSION

Diabetes mellitus is a well-known condition involving several organ systems. It can complicate the natural history of other diseases (*e.g.*, cardiovascular, neurological, oral, *etc*.), having a two-way interaction with those. [39]. In recent years, several trials have been conducted to evaluate the role of probiotics and nutraceuticals in supporting insulin therapy [40-42].

The present meta-analysis aimed to evaluate the efficacy and safety of probiotics in patients receiving treatment for T2D. Among the probiotics most frequently used as adjuncts to pharmacological therapy for T2D in the scientific literature are *Lacticaseibacillus rhamnosus*, *Limosilactobacillus reuteri*, *Akkermansia muciniphila*, and members of the *Bifidobacteriaceae* family [23].

In the selected clinical trials, the most frequently represented probiotics were *Lactobacillus acidophilus* (7/9) and *Lacticaseibacillus casei* (5/9), *Bifidobacterium bifidum* (4/9), and *Streptococcus salivarius* spp. *thermophilus* and *Bifidobacterium longum* (3/9). This study evaluated three parameters—FPG, HbA1c, and insulin concentration—and conducted three separate meta-analyses. At the conclusion of the analysis, only three studies showed a significant decrease in FPG, three studies in HbA1c, and two studies in insulin concentration. Probiotic administration exerted a positive effect on patients with T2D, as clinical improvements were reported by the end of the studies, or the measured parameters remained stable.

Our meta-analysis, based on the reviewed clinical trials, suggests that specific probiotics may be effective in supporting the treatment of T2D. Although the findings are derived from only nine clinical trials, each with relatively small sample sizes and heterogeneous patient populations, they highlight the need for further investigation into the efficacy of supplemental therapies in diabetes management.

Probiotics perform various functions that influence the host's health by reducing the inflammatory status associated with several diseases, including T2D. Based on these findings and the data obtained from this meta-analysis, probiotics could be recommended as an adjunct to standard therapy for T2D to potentially improve clinical outcomes [43].

Nevertheless, further research is required to confirm the long-term safety and efficacy of probiotic supplementation.

## CONCLUSION

In conclusion, this meta-analysis included 646 patients with T2D to evaluate the efficacy and safety of probiotic supplementation. Clinical outcomes improved in the experimental groups receiving probiotics, whereas those in the placebo groups remained unchanged. These findings support the use of probiotics as an adjunct to standard treatments for T2D.

However, the use of probiotics in T2D treatment still has certain limitations, primarily due to the small number of current trials, variability in probiotic selection, and differences in enrolled patient populations. Consequently, this limited evidence does not allow for broad generalization of the results.

Therefore, further research is encouraged to clarify the potential clinical role of specific probiotic strains in improving the management and treatment of T2D, either alone or as an adjunct to conventional therapies.

## Figures and Tables

**Fig. (1):**
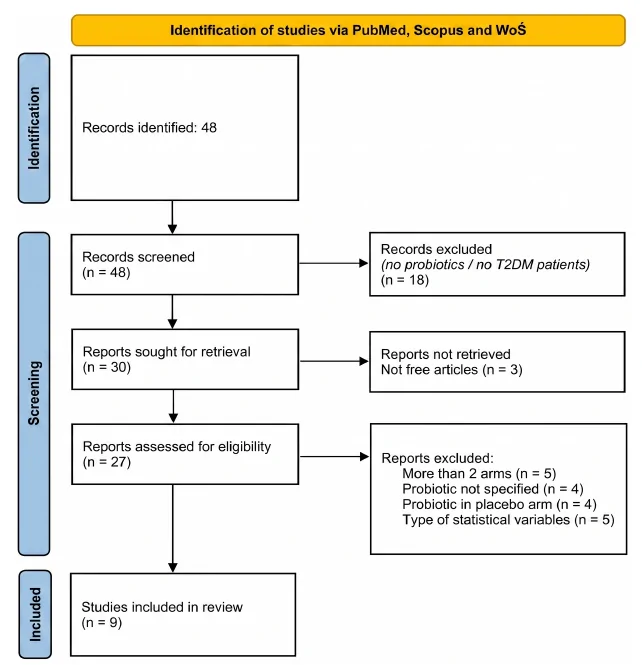
PRISMA flow diagram of study screening and selection.

**Fig. (2):**
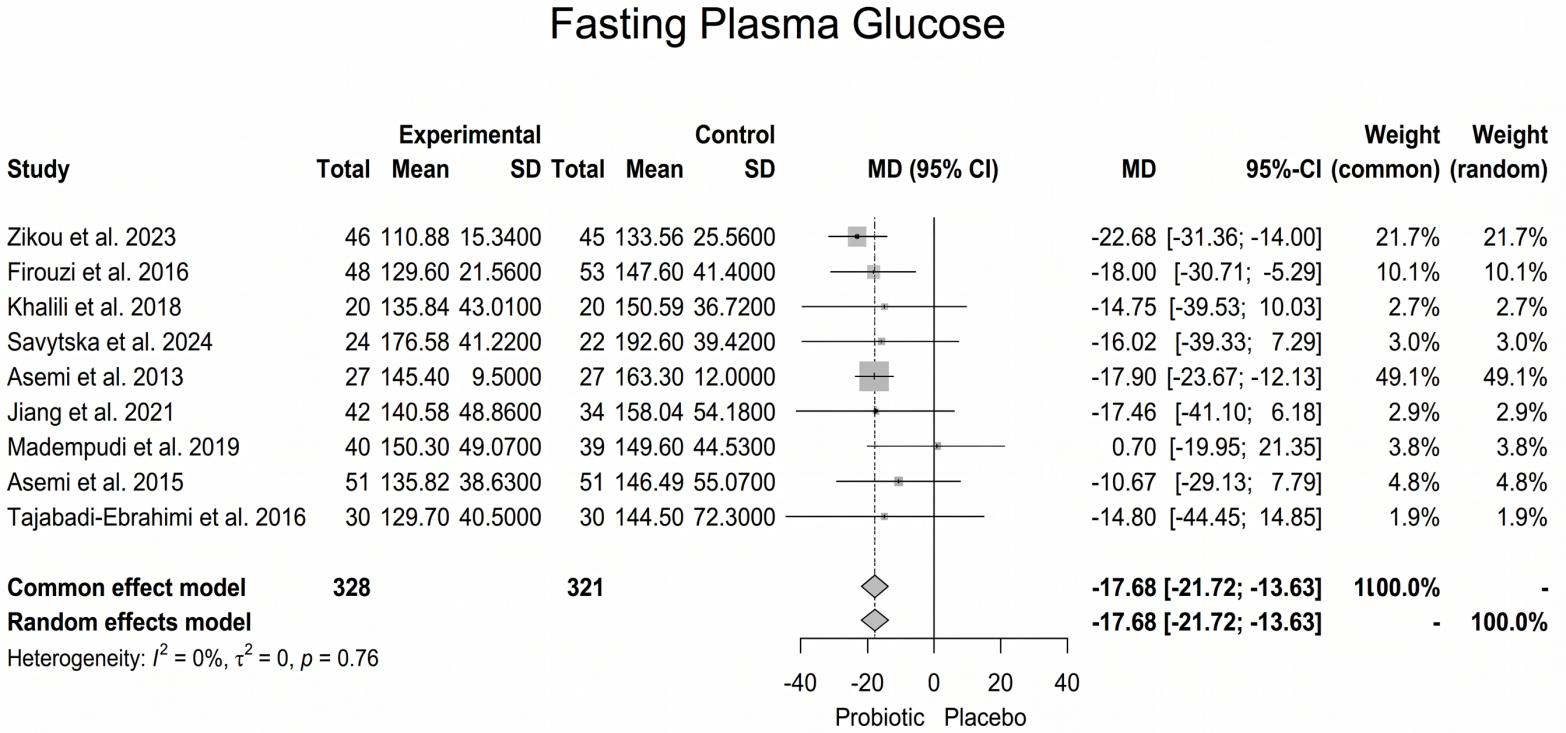
Forest plot illustrating the changes in fasting plasma glucose levels between the experimental and control groups.

**Fig. (3):**
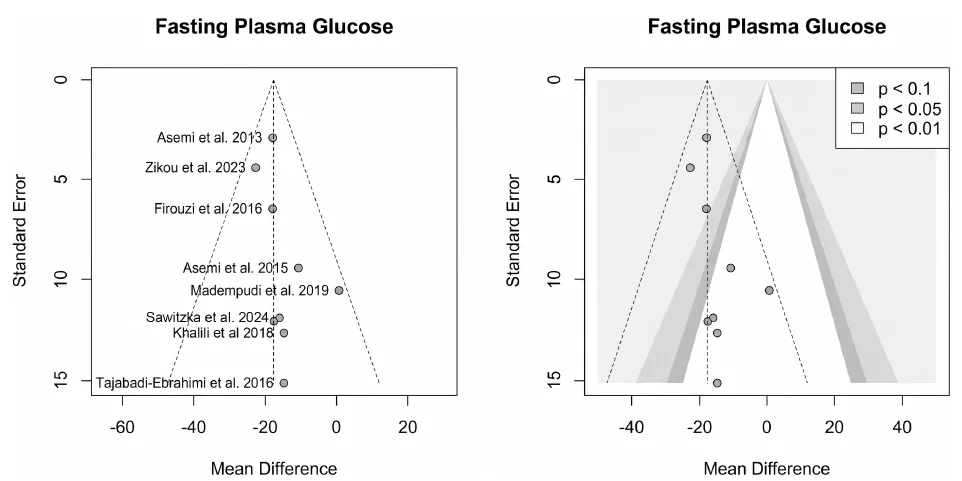
**Left:** Funnel plot of studies assessing fasting plasma glucose. **Right:** Contour-enhanced funnel plot of studies assessing fasting plasma glucose. In both plots, the x-axis and y-axis represent the mean difference and the standard error.

**Fig. (4):**
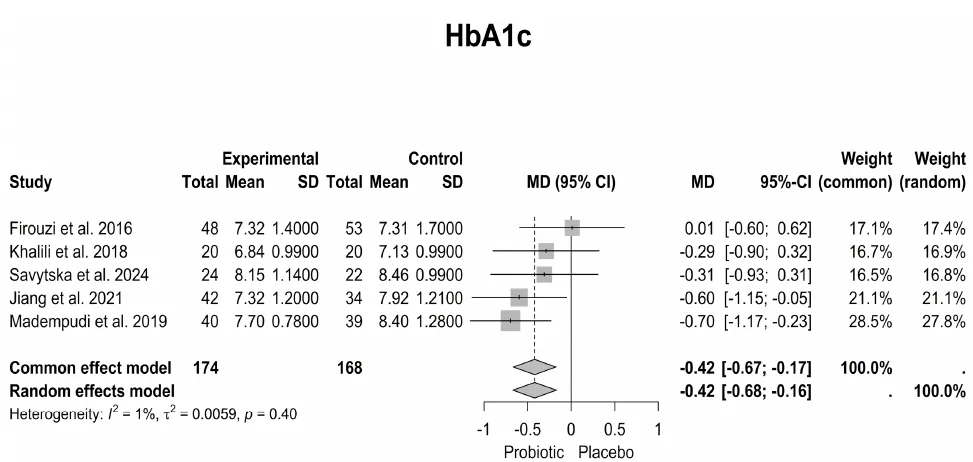
Forest plot about the variation of HbA1c in the Experimental and control arms.

**Fig. (5):**
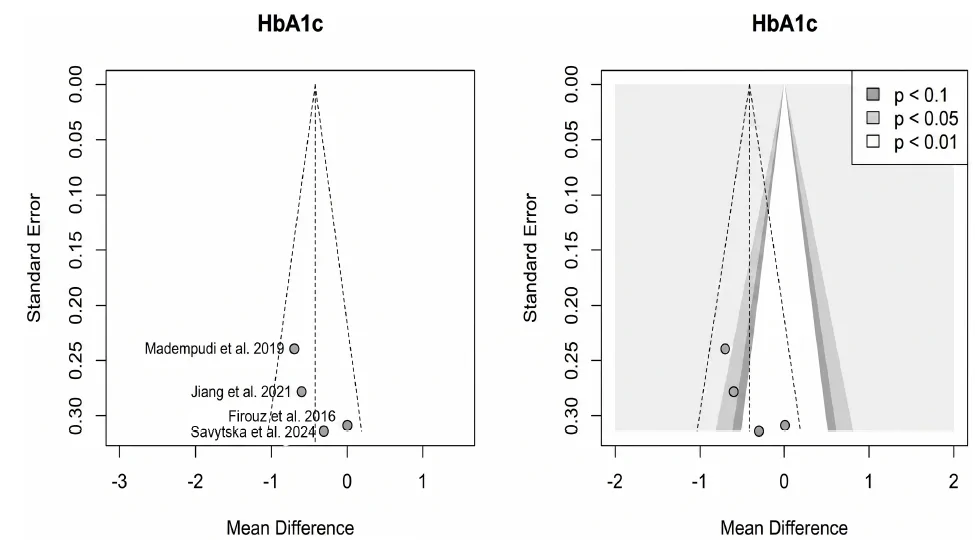
**Left:** Funnel plot of studies assessing HbA1c. **Right:** Contour-enhanced funnel plot of studies assessing HbA1c. In both plots, the x-axis and y-axis represent the mean difference and the standard error.

**Fig. (6):**
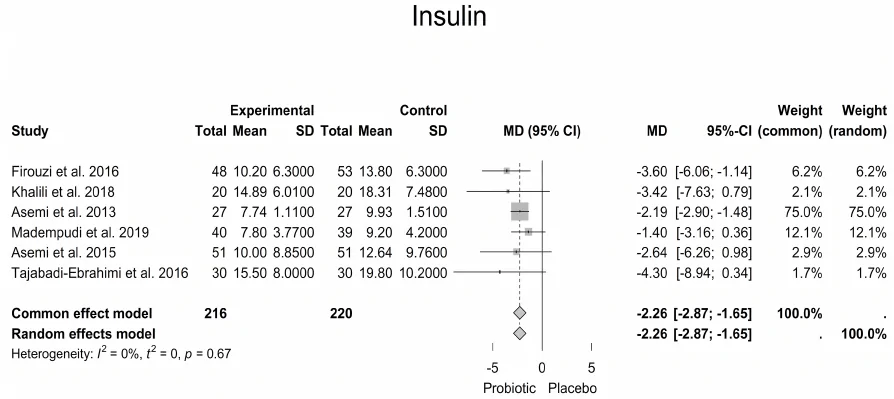
Forest plot illustrating the variations of insulin levels in the Experimental and control arms.

**Fig. (7):**
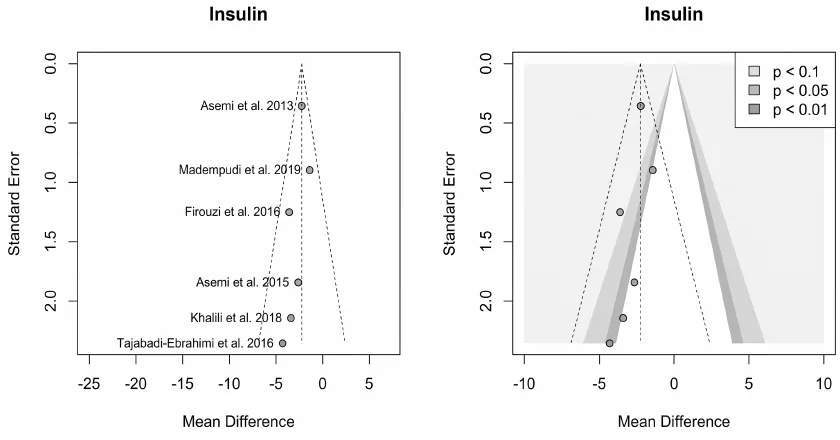
**Left:** Funnel plot about insulin. **Right:** Contour-enhanced funnel plot about insulin. In both plots, the x-axis and y-axis represent the mean difference and the standard error.

**Table 1:** Criteria for the diagnosis of diabetes.

**Parameters**	**Values**
HbA1c	≥ 6,5% (≥ 48 mmol/mol)
Fasting Plasma Glucose (FPG)	≥ 126 mg/dL (≥ 7.0 mmol/L)
during the 75 g Oral Glucose Tolerance Test (OGTT)	Plasma Glucose at 2 hours ≥ 200 mg/dL (≥ 11.1 mmol/L)
Random plasma glucose	≥ 200 mg/dL (≥ 11.1 mmol/L) *in patients with symptoms of hyperglycemia or hyperglycemic crisis*

## Data Availability

All data generated or analyzed during this study are included in this published article.

## LIST OF ABBREVIATIONS

ADA: American Diabetes Association
FPG: Fasting Plasma Glucose
OGTT: Oral Glucose Tolerance Test
PRISMA: Preferred Reporting Items for Systematic Reviews and Meta-Analysis
T2D: Type 2 Diabetes
WHO: World Health Organization

## STANDARDS OF REPORTING

PRISMA guidelines and methodologies were followed.

## SUPPLEMENTARY MATERIAL

PRISMA checklist is available as supplementary material on the publisher’s website along with the published article.
