# Supplementary material for: Probiotic Supplementation and its Potential Benefits in Type 2 Diabetes: A Meta-analysis
Source: Endocr Metab Immune Disord Drug Targets. 2026 Apr 29;26:E18715303425888. doi: 10.2174/0118715303425888251212053249 (PMC13670180; doi:10.2174/0118715303425888251212053249)
Supplement: Supplementary file 1 [file EMIDDT-26-E18715303425888_SD1.pdf]

Supplementary Material

Probiotic Supplementation and Its Potential Benefits in Type 2 Diabetes: A Meta-Analysis

Marica Colella[1,2,*], Manuela Mandorino[1], Stefania Garzone[1], Maria Elena Maggiore[1], Loredana Capozzi[1,3] and Massimo Bilancia[4]

[1]Interdisciplinary Department of Medicine, Section of Microbiology and Virology, School of Medicine, University of Bari, 70124 Bari, Italy; [2]Department of Theoretical and Applied Sciences (DiSTA), eCampus University, 22060 Novedrate, Italy; [3]Istituto Zooprofilattico Sperimentale della Puglia e della Basilicata, 71121 Foggia, Italy; [4]Unit of Statistics, Department of Precision and Regenerative Medicine and Jonian Area (DiMePRe-J), University of Bari Aldo Moro, 70124 Bari, Italy

PRISMA 2020 Checklist

Section and Topic	Item #	Checklist item	Location where item is reported
**TITLE**			
Title	1	Probiotic Supplementation and Its Potential Benefits in Type 2 Diabetes Mellitus: A Meta-Analysis	1
**ABSTRACT**			
Abstract	2	Aims: This meta-analysis aims to evaluate the efficacy and safety of probiotic supplementation as an adjunct to standard therapy in patients with type 2 diabetes mellitus (T2DM), with a focus on its effects on plasma glucose, HbA1c, and insulin levels.<br>Methods: Specific keywords such as “Type 2 Diabetes”, “Probiotics”, and “Blood Sugar” were employed to identify relevant clinical and randomized controlled trials. A comprehensive search of PubMed, Scopus, and Web of Science, conducted between September 2010 and October 2024, retrieved 48 studies, of which only 9 met the predefined inclusion criteria. Studies were excluded if they did not involve patients with T2DM, failed to specify the probiotic strains used, lacked a placebo-controlled arm, were not written in English, were not freely accessible, or did not report outcomes of interest.<br>Results: Three separate meta-analyses were conducted to assess the primary and secondary outcomes of interest: plasma glucose, HbA1c, and insulin concentrations. Each analysis revealed a statistically significant reduction in glucose, HbA1c, and insulin levels in patients with T2DM following probiotic supplementation.<br>Conclusion: This meta-analysis included a total of 764 patients with T2DM, who exhibited clinical improvements by the end of the intervention period. These findings provide strong evidence supporting the use of probiotic supplementation as an adjunct to standard therapy for the management of this condition.	1
**INTRODUCTION**			
Rationale	3	More than 480 million people worldwide are currently affected by diabetes, and this number is expected to rise substantially in the coming decades (World Health Organization, 2016). Projections estimate that over 630 million individuals could be living with the condition by 2045 (International Diabetes Federation, 2017). The global healthcare costs associated with diabetes are estimated at approximately $827 billion, underscoring the immense and growing economic burden posed by this disease.[1,2]<br>The American Diabetes Association (ADA) defines diabetes mellitus as a group of metabolic disorders affecting carbohydrate metabolism. Diabetes can be classified into several clinical types, including type 1 diabetes, type 2 diabetes, gestational diabetes mellitus, and other	3-6

Section and Topic	Item #	Checklist item	Location where item is reported
		forms resulting from various etiologies, such as exocrine pancreatic disorders, genetic factors, or drug-induced causes.<br>Diagnosis should be confirmed in patients exhibiting classic signs and symptoms, including excessive thirst and/or hunger, fatigue, frequent urination, weight changes, blurred vision, tingling or numbness in the hands or feet, recurrent infections, slow-healing wounds, or manifestations of diabetic complications such as retinopathy, nephropathy, neuropathy, impotence, or acanthosis nigricans.[3]<br>Several diagnostic tests are available for type 2 diabetes; however, current guidelines prioritize methods that combine accuracy with ease of use, as summarized in Table 1.<br>Type 2 diabetes mellitus (T2DM) represents approximately 90% of all diabetes cases. It is influenced by environmental factors and host physiology, potentially leading to reduced life expectancy and severe, costly complications. Currently, no definitive cure exists for T2DM, and management typically involves long-term use of antidiabetic medications. Therefore, exploring alternative therapeutic strategies to mitigate or reverse the progression of T2DM remains a critical priority.5,6<br>T2DM is frequently associated with hypertension, oxidative stress, and dysregulated lipid metabolism. These factors, combined with inadequate diabetes management, contribute to both microvascular and macrovascular complications. Microvascular complications, which affect small blood vessels, include diabetic retinopathy, nephropathy, and neuropathy. Macrovascular complications involve larger blood vessels and encompass cerebrovascular disease, peripheral vascular disease, and coronary artery disease. Less common complications include stroke, weight gain, congestive heart failure, electrolyte imbalances, and peripheral vascular conditions such as diabetic foot.7<br>Although T2DM is most prevalent among elderly individuals, its incidence is increasing in younger populations, including children, adolescents, and young adults. This earlier onset is attributed to various causative and triggering factors, with obesity being the most prominent. Additional risk factors implicated in the pathogenesis of T2DM include physical inactivity, excessive caloric intake, reduced total energy expenditure, genetic predisposition, low socioeconomic status, lower education levels, and alterations in gut microbiota.8<br>The composition of the gut microbiota is closely associated with overall health. An imbalance in this composition, referred to as dysbiosis, has been linked to various pathologies through the promotion of a heightened inflammatory state.[9]<br>Notably, patients with T2DM have been reported to exhibit several microbiota-related alterations, including dysbiosis, increased abundance of butyrate-producing bacteria, enhanced intestinal permeability, elevated expression of microbial genes associated with oxidative stress, reduced expression of genes involved in the synthesis of specific vitamins, and higher concentrations of lipopolysaccharide (LPS).[10,11]<br>Several studies have reported alterations in the gut microbiota of diabetic patients, characterized by a decrease in Firmicutes and an increase in Bacteroidota, Proteobacteria, and potential pathogenic opportunists, including Escherichia coli, Enterocloster clostridioformis (formerly Clostridium clostridioforme), Hungatella hathewayi (formerly Clostridium hathewayi), Clostridium symbiosum, Bacteroides caccae, Thomasclavelia ramosa (formerly Clostridium ramosum), and Eggerthella spp.<br>In this context, several studies have proposed probiotics as a supportive intervention for metabolic diseases, including T2DM. Probiotics modulate the altered gut microbiota, eliciting beneficial effects in patients with these conditions. Specifically, probiotic supplementation in T2DM has been shown to improve peripheral insulin sensitivity, preserve intestinal barrier integrity, reduce systemic levels of lipopolysaccharide (LPS), and alleviate endoplasmic reticulum stress.[12]<br>Probiotic supplementation may represent a promising therapeutic approach for T2DM and related metabolic disorders. Previous studies have demonstrated that several probiotic strains, including Lacticaseibacillus rhamnosus, Akkermansia muciniphila, and members of the Bifidobacteriaceae family, confer beneficial effects on metabolic health. For instance, supplementation with Limosilactobacillus reuteri ADR-1 or ADR-3 has been shown to redu-	

Section and Topic	Item #	Checklist item	Location where item is reported
		ce glycated hemoglobin (HbA1c) and serum cholesterol levels in patients with T2DM.13 Furthermore, a negative correlation has been observed between Akkermansia muciniphila and Bifidobacteriaceae abundance and markers of inflammation, insulin resistance, and T2DM. These microorganisms produce short-chain fatty acids (SCFAs) and contribute to intestinal barrier integrity by reducing the translocation of lipopolysaccharides (LPS) into systemic circulation. 14,15 Additionally, several studies have demonstrated mutualistic interactions between different intestinal microbiota strains, such as Lacticaseibacillus rhamnosus GG and butyrate-producing bacteria, as well as specific strains of Bifidobacterium (e.g., Ba8145) and Akkermansia muciniphila.14,16<br><br>Although exogenous probiotics are generally beneficial to human health, they may, in some cases, affect the intestinal environment by competing with endogenous gut microbiota.17 Bacterial infections and antimicrobial resistance represent critical safety concerns associated with probiotic use. Studies have indicated that supplementation with Lactobacillus, particularly Lacticaseibacillus rhamnosus, may induce bacterial translocation into the bloodstream, potentially resulting in bacteremia.18,19 Moreover, members of the Bifidobacteriaceae and Lactobacillaceae families have the potential to transfer antibiotic resistance genes to intestinal commensal pathogens.20 Therefore, it is essential to carefully evaluate the risk-benefit ratio prior to recommending probiotics as adjunctive therapy for patients with diabetes and associated conditions.21	
Objectives	4	The objective of this meta-analysis was to systematically review and compare all available clinical and randomized controlled trials assessing the efficacy of various probiotics as adjuncts to pharmacological treatment in T2DM. Specifically, we evaluated changes in fasting plasma glucose, HbA1c, and insulin concentrations.	6
**METHODS**			
Eligibility criteria	5	We included all studies meeting the following inclusion criteria:<br>1. Randomized controlled trials or clinical trials<br>2. Comparison between probiotics and placebo<br>3. Patients diagnosed with T2DM undergoing pharmacological treatment<br>4. Studies reporting at least one of the clinical outcomes of interest<br>5. Free full-text articles<br>6. Articles published in English<br><br>Conversely, we excluded studies that met any of the following exclusion criteria:<br>1. without a placebo group / where in the "placebo group" were administered probiotics<br>2. probiotic microorganism not specified<br>3. with more than two arms of intervention<br>4. with patients with prediabetes or other forms of diabetes<br>5. not free articles<br>6. studies reported as abstracts or conference presentation<br>7. do not report any outcome of interests	7
Information sources	6	The literature search was performed on PubMed, Scopus, and Web of Science, covering the period from September 2010 to October 2024. The keywords used were "Type 2 diabetes mellitus" AND "Probiotics" AND "Blood sugar," with filters applied for "Humans," "Clinical trials," and "Randomized controlled trials." This search retrieved 48 results, of which only 9 studies met the inclusion criteria and were included in the meta-analysis.	7
Search strategy	7	The literature search was performed on PubMed, Scopus, and Web of Science, covering the period from September 2010 to October 2024. The keywords used were "Type 2 diabetes mellitus" AND "Probiotics" AND "Blood sugar," with filters applied for "Humans," "Clinical trials," and "Randomized controlled trials." This search retrieved 48 results, of which only 9	7

Section and Topic	Item #	Checklist item	Location where item is reported
		studies met the inclusion criteria and were included in the meta-analysis.	
Selection process	8	The literature search was performed on PubMed, Scopus, and Web of Science, covering the period from September 2010 to October 2024. The keywords used were "Type 2 diabetes mellitus" AND "Probiotics" AND "Blood sugar," with filters applied for "Humans," "Clinical trials," and "Randomized controlled trials." This search retrieved 48 results, of which only 9 studies met the inclusion criteria and were included in the meta-analysis.	7
Data collection process	9	The literature search was performed on PubMed, Scopus, and Web of Science, covering the period from September 2010 to October 2024. The keywords used were "Type 2 diabetes mellitus" AND "Probiotics" AND "Blood sugar," with filters applied for "Humans," "Clinical trials," and "Randomized controlled trials." This search retrieved 48 results, of which only 9 studies met the inclusion criteria and were included in the meta-analysis.	7
Data items	10a	The primary objective of this study was to evaluate the effect of probiotic supplementation on plasma glucose concentrations, compared to baseline levels, in patients with T2DM. Secondary outcomes included the mean changes in HbA1c percentage and insulin concentration following probiotic administration. Patients who experienced adverse reactions to probiotics were excluded from all studies included in this meta-analysis.	7-8
	10b	The primary objective of this study was to evaluate the effect of probiotic supplementation on plasma glucose concentrations, compared to baseline levels, in patients with T2DM. Secondary outcomes included the mean changes in HbA1c percentage and insulin concentration following probiotic administration. Patients who experienced adverse reactions to probiotics were excluded from all studies included in this meta-analysis.	7-8
Study risk of bias assessment	11	The overall mean difference (MD) effect was estimated using both fixed-effects and random-effects models, with results presented in a forest plot.22 Heterogeneity was assessed using the I^2 statistic, which quantifies the proportion of variability attributable to heterogeneity rather than to sampling error.23 Publication bias was assessed using funnel plots.24 All analyses were conducted in R version 4.4.2, using the Meta package (version 8.0-1).25,26	8
Effect measures	12	The overall mean difference (MD) effect was estimated using both fixed-effects and random-effects models, with results presented in a forest plot.22 Heterogeneity was assessed using the I^2 statistic, which quantifies the proportion of variability attributable to heterogeneity rather than to sampling error.23 Publication bias was assessed using funnel plots.24 All analyses were conducted in R version 4.4.2, using the Meta package (version 8.0-1).25,26	8
Synthesis methods	13a	The overall mean difference (MD) effect was estimated using both fixed-effects and random-effects models, with results presented in a forest plot.22 Heterogeneity was assessed using the I^2 statistic, which quantifies the proportion of variability attributable to heterogeneity rather than to sampling error.23 Publication bias was assessed using funnel plots.24 All analyses were conducted in R version 4.4.2, using the Meta package (version 8.0-1).25,26	8
	13b	The overall mean difference (MD) effect was estimated using both fixed-effects and random-effects models, with results presented in a forest plot.22 Heterogeneity was assessed using the I^2 statistic, which quantifies the proportion of variability attributable to heterogeneity rather than to sampling error.23 Publication bias was assessed using funnel plots.24 All analyses were conducted in R version 4.4.2, using the Meta package (version 8.0-1).25,26	8
	13c	The overall mean difference (MD) effect was estimated using both fixed-effects and random-effects models, with results presented in a forest plot.22 Heterogeneity was assessed using the I^2 statistic, which quantifies the proportion of variability attributable to heterogeneity rather than to sampling error.23 Publication bias was assessed using funnel plots.24 All analyses were conducted in R version 4.4.2, using the Meta package (version 8.0-1).25,26	8
	13d	The overall mean difference (MD) effect was estimated using both fixed-effects and random-effects models, with results presented in a forest plot.22 Heterogeneity was assessed using the I^2 statistic, which quantifies the proportion of variability attributable to heterogeneity rather than to sampling error.23 Publication bias was assessed using funnel plots.24 All analyses were conducted in R version 4.4.2, using the Meta package (version 8.0-1).25,26	8
	13e	The overall mean difference (MD) effect was estimated using both fixed-effects and random-effects models, with results presented in a forest plot.22 Heterogeneity was assessed	8

Section and Topic	Item #	Checklist item	Location where item is reported
		using the I^2 statistic, which quantifies the proportion of variability attributable to heterogeneity rather than to sampling error.23 Publication bias was assessed using funnel plots.24 All analyses were conducted in R version 4.4.2, using the Meta package (version 8.0-1).25,26	
	13f	The overall mean difference (MD) effect was estimated using both fixed-effects and random-effects models, with results presented in a forest plot.22 Heterogeneity was assessed using the I^2 statistic, which quantifies the proportion of variability attributable to heterogeneity rather than to sampling error.23 Publication bias was assessed using funnel plots.24 All analyses were conducted in R version 4.4.2, using the Meta package (version 8.0-1).25,26	8
Reporting bias assessment	14	The overall mean difference (MD) effect was estimated using both fixed-effects and random-effects models, with results presented in a forest plot.22 Heterogeneity was assessed using the I^2 statistic, which quantifies the proportion of variability attributable to heterogeneity rather than to sampling error.23 Publication bias was assessed using funnel plots.24 All analyses were conducted in R version 4.4.2, using the Meta package (version 8.0-1).25,26	8
Certainty assessment	15	The overall mean difference (MD) effect was estimated using both fixed-effects and random-effects models, with results presented in a forest plot.22 Heterogeneity was assessed using the I^2 statistic, which quantifies the proportion of variability attributable to heterogeneity rather than to sampling error.23 Publication bias was assessed using funnel plots.24 All analyses were conducted in R version 4.4.2, using the Meta package (version 8.0-1).25,26	8
**RESULTS**			
Study selection	16a	As illustrated in Figure 1, the search strategy identified 48 studies; however, applying our eligibility criteria resulted in the inclusion of only 9 studies, which were fully analyzed.<br><br>The total population across the nine clinical trials comprised 646 patients with T2DM: 325 in the experimental (probiotic) group and 321 in the control (placebo) group. Patient characteristics were comparable between groups, with ages ranging from 18 to 75 years. In all included trials, the following parameters were evaluated in both arms: fasting plasma glucose (FPG, mg/dL), HbA1c (%), and insulin concentration (μIU/mL). Among the selected studies, all assessed FPG, seven evaluated insulin levels, and six measured HbA1c.	8
	16b	As illustrated in Figure 1, the search strategy identified 48 studies; however, applying our eligibility criteria resulted in the inclusion of only 9 studies, which were fully analyzed.	8
Study characteristics	17	The total population across the nine clinical trials comprised 646 patients with T2DM: 325 in the experimental (probiotic) group and 321 in the control (placebo) group. Patient characteristics were comparable between groups, with ages ranging from 18 to 75 years. In all included trials, the following parameters were evaluated in both arms: fasting plasma glucose (FPG, mg/dL), HbA1c (%), and insulin concentration (μIU/mL). Among the selected studies, all assessed FPG, seven evaluated insulin levels, and six measured HbA1c.<br><br>The mean difference between the experimental and control groups (common effect model: MD = -17.67; 95% CI: -21.72 to -13.63; $p < 0.0001$; $I^2 = 0\%$; p for heterogeneity = 0.76; Figure 23) indicates a significant reduction in fasting plasma glucose (FPG) levels in the experimental group following probiotic supplementation. No heterogeneity was observed among the studies, as reflected by the I^2 value of 0.0% (95% CI: 0.0% to 64.8%). Additionally, a funnel plot analysis of these nine studies (Figure 3 - Left) revealed an overall symmetrical distribution, suggesting no significant publication bias (Figure 3 - Right). However, several studies exhibited high standard errors, such as those by Tajabadi-Ebrahimi et al. (2016), Khalili et al. (2018), Jiang et al. (2024), and Savytska et al. (2024).28,29,30,31 We generated a contour-enhanced funnel plot (Figure 3 - Left), which helped clarify the statistical significance. The studies with a significant effect size ($p < 0.01$) were identified as three: Asemi et al. (2013), Zikou et al. (2023), and Firouzi et al. (2016).<br><br>At the end of the treatment, the probiotic groups showed a significantly greater reduction in mean difference compared to the placebo group (common effect model: MD = -0.42; 95% CI: -0.67 to -0.17; $p = 0.0009$; $I^2 = 0.5\%$; p for heterogeneity = 0.40; Figure 4). This analysis included data from five studies, as not all nine trials reported this parameter. The funnel plot (Figure 5 - Left) demonstrated an overall symmetrical distribution. A very low degree of heterogeneity was observed ($I^2 = 0.5\%$; 95% CI: 0.0% to 79.3%).30,31,32,33,35<br><br>However, the data also exhibited an asymmetrical pattern, which prompted the generation	8-13

Section and Topic	Item #	Checklist item	Location where item is reported
		of a contour-enhanced funnel plot (Figure 5 - Right). This analysis identified that the studies showing a significant effect ($p < 0.01$) were those by Madempudi et al. (2019) and Jiang et al. (2021).<br><br>The mean difference indicates a significant effect between groups at the end of the study (common effect model: MD = -2.26; 95% CI: -2.87 to -1.64; $p < 0.0001$; $I^2 = 0\%$; p for heterogeneity = 0.67; Figure 6). This analysis included data from six studies. The funnel plot (Figure 7 - Left) shows that all studies fall within the expected funnel shape, suggesting the absence of publication bias. A low degree of heterogeneity was observed ($I^2 = 0.0\%$; 95% CI: 0.0% to 74.6%). We also generated a contour-enhanced funnel plot (Figure 7 - Right), which revealed that only two studies (Asemi et al., 2015 and Firouzi et al., 2016) presented a p-value lower than 0.01 ($p < 0.01$).	
Risk of bias in studies	18	The total population across the nine clinical trials comprised 646 patients with T2DM: 325 in the experimental (probiotic) group and 321 in the control (placebo) group. Patient characteristics were comparable between groups, with ages ranging from 18 to 75 years. In all included trials, the following parameters were evaluated in both arms: fasting plasma glucose (FPG, mg/dL), HbA1c (%), and insulin concentration (µIU/mL). Among the selected studies, all assessed FPG, seven evaluated insulin levels, and six measured HbA1c.<br><br>The mean difference between the experimental and control groups (common effect model: MD = -17.67; 95% CI: -21.72 to -13.63; $p < 0.0001$; $I^2 = 0\%$; p for heterogeneity = 0.76; Figure 23) indicates a significant reduction in fasting plasma glucose (FPG) levels in the experimental group following probiotic supplementation. No heterogeneity was observed among the studies, as reflected by the I^2 value of 0.0% (95% CI: 0.0% to 64.8%). Additionally, a funnel plot analysis of these nine studies (Figure 3 - Left) revealed an overall symmetrical distribution, suggesting no significant publication bias (Figure 3 - Right). However, several studies exhibited high standard errors, such as those by Tajabadi-Ebrahimi et al. (2016), Khalili et al. (2018), Jiang et al. (2024), and Savytska et al. (2024).28,29,30,31 We generated a contour-enhanced funnel plot (Figure 3 - Left), which helped clarify the statistical significance. The studies with a significant effect size ($p < 0.01$) were identified as three: Asemi et al. (2013), Zikou et al. (2023), and Firouzi et al. (2016).<br><br>At the end of the treatment, the probiotic groups showed a significantly greater reduction in mean difference compared to the placebo group (common effect model: MD = -0.42; 95% CI: -0.67 to -0.17; $p = 0.0009$; $I^2 = 0.5\%$; p for heterogeneity = 0.40; Figure 4). This analysis included data from five studies, as not all nine trials reported this parameter. The funnel plot (Figure 5 - Left) demonstrated an overall symmetrical distribution. A very low degree of heterogeneity was observed ($I^2 = 0.5\%$; 95% CI: 0.0% to 79.3%).30,31,32,33,35<br><br>However, the data also exhibited an asymmetrical pattern, which prompted the generation of a contour-enhanced funnel plot (Figure 5 - Right). This analysis identified that the studies showing a significant effect ($p < 0.01$) were those by Madempudi et al. (2019) and Jiang et al. (2021).<br><br>The mean difference indicates a significant effect between groups at the end of the study (common effect model: MD = -2.26; 95% CI: -2.87 to -1.64; $p < 0.0001$; $I^2 = 0\%$; p for heterogeneity = 0.67; Figure 6). This analysis included data from six studies. The funnel plot (Figure 7 - Left) shows that all studies fall within the expected funnel shape, suggesting the absence of publication bias. A low degree of heterogeneity was observed ($I^2 = 0.0\%$; 95% CI: 0.0% to 74.6%). We also generated a contour-enhanced funnel plot (Figure 7 - Right), which revealed that only two studies (Asemi et al., 2015 and Firouzi et al., 2016) presented a p-value lower than 0.01 ($p < 0.01$).	8-13
Results of individual studies	19	The total population across the nine clinical trials comprised 646 patients with T2DM: 325 in the experimental (probiotic) group and 321 in the control (placebo) group. Patient characteristics were comparable between groups, with ages ranging from 18 to 75 years. In all included trials, the following parameters were evaluated in both arms: fasting plasma glucose (FPG, mg/dL), HbA1c (%), and insulin concentration (µIU/mL). Among the selected studies, all assessed FPG, seven evaluated insulin levels, and six measured HbA1c.<br><br>The mean difference between the experimental and control groups (common effect model: MD = -17.67; 95% CI: -21.72 to -13.63; $p < 0.0001$; $I^2 = 0\%$; p for heterogeneity = 0.76; Fi-	8-13

Section and Topic	Item #	Checklist item	Location where item is reported
		gure 23) indicates a significant reduction in fasting plasma glucose (FPG) levels in the experimental group following probiotic supplementation. No heterogeneity was observed among the studies, as reflected by the I² value of 0.0% (95% CI: 0.0% to 64.8%). Additionally, a funnel plot analysis of these nine studies (Figure 3 - Left) revealed an overall symmetrical distribution, suggesting no significant publication bias (Figure 3 - Right). However, several studies exhibited high standard errors, such as those by Tajabadi-Ebrahimi et al. (2016), Khalili et al. (2018), Jiang et al. (2024), and Savytska et al. (2024).28,29,30,31 We generated a contour-enhanced funnel plot (Figure 3 - Left), which helped clarify the statistical significance. The studies with a significant effect size ($p < 0.01$) were identified as three: Asemi et al. (2013), Zikou et al. (2023), and Firouzi et al. (2016).<br><br>At the end of the treatment, the probiotic groups showed a significantly greater reduction in mean difference compared to the placebo group (common effect model: MD = -0.42; 95% CI: -0.67 to -0.17; p = 0.0009; I^2 = 0.5%; p for heterogeneity = 0.40; Figure 4). This analysis included data from five studies, as not all nine trials reported this parameter. The funnel plot (Figure 5 - Left) demonstrated an overall symmetrical distribution. A very low degree of heterogeneity was observed (I^2 = 0.5%; 95% CI: 0.0% to 79.3%).30,31,32,33,35<br><br>However, the data also exhibited an asymmetrical pattern, which prompted the generation of a contour-enhanced funnel plot (Figure 5 - Right). This analysis identified that the studies showing a significant effect ($p < 0.01$) were those by Madempudi et al. (2019) and Jiang et al. (2021).<br><br>The mean difference indicates a significant effect between groups at the end of the study (common effect model: MD = -2.26; 95% CI: -2.87 to -1.64; $p < 0.0001$; I^2 = 0%; p for heterogeneity = 0.67; Figure 6). This analysis included data from six studies. The funnel plot (Figure 7 - Left) shows that all studies fall within the expected funnel shape, suggesting the absence of publication bias. A low degree of heterogeneity was observed (I^2 = 0.0%; 95% CI: 0.0% to 74.6%). We also generated a contour-enhanced funnel plot (Figure 7 - Right), which revealed that only two studies (Asemi et al., 2015 and Firouzi et al., 2016) presented a p-value lower than 0.01 ($p < 0.01$).	
Results of syntheses	20a	The total population across the nine clinical trials comprised 646 patients with T2DM: 325 in the experimental (probiotic) group and 321 in the control (placebo) group. Patient characteristics were comparable between groups, with ages ranging from 18 to 75 years. In all included trials, the following parameters were evaluated in both arms: fasting plasma glucose (FPG, mg/dL), HbA1c (%), and insulin concentration (µIU/mL). Among the selected studies, all assessed FPG, seven evaluated insulin levels, and six measured HbA1c.<br><br>The mean difference between the experimental and control groups (common effect model: MD = -17.67; 95% CI: -21.72 to -13.63; $p < 0.0001$; I^2 = 0%; p for heterogeneity = 0.76; Figure 23) indicates a significant reduction in fasting plasma glucose (FPG) levels in the experimental group following probiotic supplementation. No heterogeneity was observed among the studies, as reflected by the I² value of 0.0% (95% CI: 0.0% to 64.8%). Additionally, a funnel plot analysis of these nine studies (Figure 3 - Left) revealed an overall symmetrical distribution, suggesting no significant publication bias (Figure 3 - Right). However, several studies exhibited high standard errors, such as those by Tajabadi-Ebrahimi et al. (2016), Khalili et al. (2018), Jiang et al. (2024), and Savytska et al. (2024).28,29,30,31 We generated a contour-enhanced funnel plot (Figure 3 - Left), which helped clarify the statistical significance. The studies with a significant effect size ($p < 0.01$) were identified as three: Asemi et al. (2013), Zikou et al. (2023), and Firouzi et al. (2016).<br><br>At the end of the treatment, the probiotic groups showed a significantly greater reduction in mean difference compared to the placebo group (common effect model: MD = -0.42; 95% CI: -0.67 to -0.17; p = 0.0009; I^2 = 0.5%; p for heterogeneity = 0.40; Figure 4). This analysis included data from five studies, as not all nine trials reported this parameter. The funnel plot (Figure 5 - Left) demonstrated an overall symmetrical distribution. A very low degree of heterogeneity was observed (I^2 = 0.5%; 95% CI: 0.0% to 79.3%).30,31,32,33,35<br><br>However, the data also exhibited an asymmetrical pattern, which prompted the generation of a contour-enhanced funnel plot (Figure 5 - Right). This analysis identified that the studies showing a significant effect ($p < 0.01$) were those by Madempudi et al. (2019) and Jiang et	8-13

Section and Topic	Item #	Checklist item	Location where item is reported
		al. (2021).<br><br>The mean difference indicates a significant effect between groups at the end of the study (common effect model: MD = -2.26; 95% CI: -2.87 to -1.64; $p < 0.0001$; $I^2 = 0\%$; p for heterogeneity = 0.67; Figure 6). This analysis included data from six studies. The funnel plot (Figure 7 - Left) shows that all studies fall within the expected funnel shape, suggesting the absence of publication bias. A low degree of heterogeneity was observed ($I^2 = 0.0\%$; 95% CI: 0.0% to 74.6%). We also generated a contour-enhanced funnel plot (Figure 7 - Right), which revealed that only two studies (Asemi et al., 2015 and Firouzi et al., 2016) presented a p-value lower than 0.01 ($p < 0.01$).	
	20b	The total population across the nine clinical trials comprised 646 patients with T2DM: 325 in the experimental (probiotic) group and 321 in the control (placebo) group. Patient characteristics were comparable between groups, with ages ranging from 18 to 75 years. In all included trials, the following parameters were evaluated in both arms: fasting plasma glucose (FPG, mg/dL), HbA1c (%), and insulin concentration (µIU/mL). Among the selected studies, all assessed FPG, seven evaluated insulin levels, and six measured HbA1c.<br><br>The mean difference between the experimental and control groups (common effect model: MD = -17.67; 95% CI: -21.72 to -13.63; $p < 0.0001$; $I^2 = 0\%$; p for heterogeneity = 0.76; Figure 23) indicates a significant reduction in fasting plasma glucose (FPG) levels in the experimental group following probiotic supplementation. No heterogeneity was observed among the studies, as reflected by the I^2 value of 0.0% (95% CI: 0.0% to 64.8%). Additionally, a funnel plot analysis of these nine studies (Figure 3 - Left) revealed an overall symmetrical distribution, suggesting no significant publication bias (Figure 3 - Right). However, several studies exhibited high standard errors, such as those by Tajabadi-Ebrahimi et al. (2016), Khalili et al. (2018), Jiang et al. (2024), and Savytska et al. (2024).[28,29,30,31] We generated a contour-enhanced funnel plot (Figure 3 - Left), which helped clarify the statistical significance. The studies with a significant effect size ($p < 0.01$) were identified as three: Asemi et al. (2013), Zikou et al. (2023), and Firouzi et al. (2016).<br><br>At the end of the treatment, the probiotic groups showed a significantly greater reduction in mean difference compared to the placebo group (common effect model: MD = -0.42; 95% CI: -0.67 to -0.17; $p = 0.0009$; $I^2 = 0.5\%$; p for heterogeneity = 0.40; Figure 4). This analysis included data from five studies, as not all nine trials reported this parameter. The funnel plot (Figure 5 - Left) demonstrated an overall symmetrical distribution. A very low degree of heterogeneity was observed ($I^2 = 0.5\%$; 95% CI: 0.0% to 79.3%).[30,31,32,33,35]<br><br>However, the data also exhibited an asymmetrical pattern, which prompted the generation of a contour-enhanced funnel plot (Figure 5 - Right). This analysis identified that the studies showing a significant effect ($p < 0.01$) were those by Madempudi et al. (2019) and Jiang et al. (2021).<br><br>The mean difference indicates a significant effect between groups at the end of the study (common effect model: MD = -2.26; 95% CI: -2.87 to -1.64; $p < 0.0001$; $I^2 = 0\%$; p for heterogeneity = 0.67; Figure 6). This analysis included data from six studies. The funnel plot (Figure 7 - Left) shows that all studies fall within the expected funnel shape, suggesting the absence of publication bias. A low degree of heterogeneity was observed ($I^2 = 0.0\%$; 95% CI: 0.0% to 74.6%). We also generated a contour-enhanced funnel plot (Figure 7 - Right), which revealed that only two studies (Asemi et al., 2015 and Firouzi et al., 2016) presented a p-value lower than 0.01 ($p < 0.01$).	8-13
	20c	The total population across the nine clinical trials comprised 646 patients with T2DM: 325 in the experimental (probiotic) group and 321 in the control (placebo) group. Patient characteristics were comparable between groups, with ages ranging from 18 to 75 years. In all included trials, the following parameters were evaluated in both arms: fasting plasma glucose (FPG, mg/dL), HbA1c (%), and insulin concentration (µIU/mL). Among the selected studies, all assessed FPG, seven evaluated insulin levels, and six measured HbA1c.<br><br>The mean difference between the experimental and control groups (common effect model: MD = -17.67; 95% CI: -21.72 to -13.63; $p < 0.0001$; $I^2 = 0\%$; p for heterogeneity = 0.76; Figure 23) indicates a significant reduction in fasting plasma glucose (FPG) levels in the experimental group following probiotic supplementation. No heterogeneity was observed among	8-13

Section and Topic	Item #	Checklist item	Location where item is reported
		the studies, as reflected by the I^2 value of 0.0% (95% CI: 0.0% to 64.8%). Additionally, a funnel plot analysis of these nine studies (Figure 3 - Left) revealed an overall symmetrical distribution, suggesting no significant publication bias (Figure 3 - Right). However, several studies exhibited high standard errors, such as those by Tajabadi-Ebrahimi et al. (2016), Khalili et al. (2018), Jiang et al. (2024), and Savytska et al. (2024).[28,29,30,31] We generated a contour-enhanced funnel plot (Figure 3 - Left), which helped clarify the statistical significance. The studies with a significant effect size ($p < 0.01$) were identified as three: Asemi et al. (2013), Zikou et al. (2023), and Firouzi et al. (2016).<br><br>At the end of the treatment, the probiotic groups showed a significantly greater reduction in mean difference compared to the placebo group (common effect model: MD = -0.42; 95% CI: -0.67 to -0.17; $p = 0.0009$; $I^2 = 0.5\%$; p for heterogeneity = 0.40; Figure 4). This analysis included data from five studies, as not all nine trials reported this parameter. The funnel plot (Figure 5 - Left) demonstrated an overall symmetrical distribution. A very low degree of heterogeneity was observed ($I^2 = 0.5\%$; 95% CI: 0.0% to 79.3%).[30,31,32,33,35]<br><br>However, the data also exhibited an asymmetrical pattern, which prompted the generation of a contour-enhanced funnel plot (Figure 5 - Right). This analysis identified that the studies showing a significant effect ($p < 0.01$) were those by Madempudi et al. (2019) and Jiang et al. (2021).<br><br>The mean difference indicates a significant effect between groups at the end of the study (common effect model: MD = -2.26; 95% CI: -2.87 to -1.64; $p < 0.0001$; $I^2 = 0\%$; p for heterogeneity = 0.67; Figure 6). This analysis included data from six studies. The funnel plot (Figure 7 - Left) shows that all studies fall within the expected funnel shape, suggesting the absence of publication bias. A low degree of heterogeneity was observed ($I^2 = 0.0\%$; 95% CI: 0.0% to 74.6%). We also generated a contour-enhanced funnel plot (Figure 7 - Right), which revealed that only two studies (Asemi et al., 2015 and Firouzi et al., 2016) presented a p-value lower than 0.01 ($p < 0.01$).	
	20d	The total population across the nine clinical trials comprised 646 patients with T2DM: 325 in the experimental (probiotic) group and 321 in the control (placebo) group. Patient characteristics were comparable between groups, with ages ranging from 18 to 75 years. In all included trials, the following parameters were evaluated in both arms: fasting plasma glucose (FPG, mg/dL), HbA1c (%), and insulin concentration (μIU/mL). Among the selected studies, all assessed FPG, seven evaluated insulin levels, and six measured HbA1c.<br><br>The mean difference between the experimental and control groups (common effect model: MD = -17.67; 95% CI: -21.72 to -13.63; $p < 0.0001$; $I^2 = 0\%$; p for heterogeneity = 0.76; Figure 23) indicates a significant reduction in fasting plasma glucose (FPG) levels in the experimental group following probiotic supplementation. No heterogeneity was observed among the studies, as reflected by the I^2 value of 0.0% (95% CI: 0.0% to 64.8%). Additionally, a funnel plot analysis of these nine studies (Figure 3 - Left) revealed an overall symmetrical distribution, suggesting no significant publication bias (Figure 3 - Right). However, several studies exhibited high standard errors, such as those by Tajabadi-Ebrahimi et al. (2016), Khalili et al. (2018), Jiang et al. (2024), and Savytska et al. (2024).[28,29,30,31] We generated a contour-enhanced funnel plot (Figure 3 - Left), which helped clarify the statistical significance. The studies with a significant effect size ($p < 0.01$) were identified as three: Asemi et al. (2013), Zikou et al. (2023), and Firouzi et al. (2016).<br><br>At the end of the treatment, the probiotic groups showed a significantly greater reduction in mean difference compared to the placebo group (common effect model: MD = -0.42; 95% CI: -0.67 to -0.17; $p = 0.0009$; $I^2 = 0.5\%$; p for heterogeneity = 0.40; Figure 4). This analysis included data from five studies, as not all nine trials reported this parameter. The funnel plot (Figure 5 - Left) demonstrated an overall symmetrical distribution. A very low degree of heterogeneity was observed ($I^2 = 0.5\%$; 95% CI: 0.0% to 79.3%).[30,31,32,33,35]<br><br>However, the data also exhibited an asymmetrical pattern, which prompted the generation of a contour-enhanced funnel plot (Figure 5 - Right). This analysis identified that the studies showing a significant effect ($p < 0.01$) were those by Madempudi et al. (2019) and Jiang et al. (2021).<br><br>The mean difference indicates a significant effect between groups at the end of the study	8-13

Section and Topic	Item #	Checklist item	Location where item is reported
		(common effect model: MD = -2.26; 95% CI: -2.87 to -1.64; $p < 0.0001$; $I^2 = 0\%$; p for heterogeneity = 0.67; Figure 6). This analysis included data from six studies. The funnel plot (Figure 7 - Left) shows that all studies fall within the expected funnel shape, suggesting the absence of publication bias. A low degree of heterogeneity was observed ($I^2 = 0.0\%$; 95% CI: 0.0% to 74.6%). We also generated a contour-enhanced funnel plot (Figure 7 - Right), which revealed that only two studies (Asemi et al., 2015 and Firouzi et al., 2016) presented a p-value lower than 0.01 ($p < 0.01$).	
Reporting biases	21	The total population across the nine clinical trials comprised 646 patients with T2DM: 325 in the experimental (probiotic) group and 321 in the control (placebo) group. Patient characteristics were comparable between groups, with ages ranging from 18 to 75 years. In all included trials, the following parameters were evaluated in both arms: fasting plasma glucose (FPG, mg/dL), HbA1c (%), and insulin concentration (μIU/mL). Among the selected studies, all assessed FPG, seven evaluated insulin levels, and six measured HbA1c.<br><br>The mean difference between the experimental and control groups (common effect model: MD = -17.67; 95% CI: -21.72 to -13.63; $p < 0.0001$; $I^2 = 0\%$; p for heterogeneity = 0.76; Figure 23) indicates a significant reduction in fasting plasma glucose (FPG) levels in the experimental group following probiotic supplementation. No heterogeneity was observed among the studies, as reflected by the I^2 value of 0.0% (95% CI: 0.0% to 64.8%). Additionally, a funnel plot analysis of these nine studies (Figure 3 - Left) revealed an overall symmetrical distribution, suggesting no significant publication bias (Figure 3 - Right). However, several studies exhibited high standard errors, such as those by Tajabadi-Ebrahimi et al. (2016), Khalili et al. (2018), Jiang et al. (2024), and Savytska et al. (2024).28,29,30,31 We generated a contour-enhanced funnel plot (Figure 3 - Left), which helped clarify the statistical significance. The studies with a significant effect size ($p < 0.01$) were identified as three: Asemi et al. (2013), Zikou et al. (2023), and Firouzi et al. (2016).<br><br>At the end of the treatment, the probiotic groups showed a significantly greater reduction in mean difference compared to the placebo group (common effect model: MD = -0.42; 95% CI: -0.67 to -0.17; $p = 0.0009$; $I^2 = 0.5\%$; p for heterogeneity = 0.40; Figure 4). This analysis included data from five studies, as not all nine trials reported this parameter. The funnel plot (Figure 5 - Left) demonstrated an overall symmetrical distribution. A very low degree of heterogeneity was observed ($I^2 = 0.5\%$; 95% CI: 0.0% to 79.3%).30,31,32,33,35<br><br>However, the data also exhibited an asymmetrical pattern, which prompted the generation of a contour-enhanced funnel plot (Figure 5 - Right). This analysis identified that the studies showing a significant effect ($p < 0.01$) were those by Madempudi et al. (2019) and Jiang et al. (2021).<br><br>The mean difference indicates a significant effect between groups at the end of the study (common effect model: MD = -2.26; 95% CI: -2.87 to -1.64; $p < 0.0001$; $I^2 = 0\%$; p for heterogeneity = 0.67; Figure 6). This analysis included data from six studies. The funnel plot (Figure 7 - Left) shows that all studies fall within the expected funnel shape, suggesting the absence of publication bias. A low degree of heterogeneity was observed ($I^2 = 0.0\%$; 95% CI: 0.0% to 74.6%). We also generated a contour-enhanced funnel plot (Figure 7 - Right), which revealed that only two studies (Asemi et al., 2015 and Firouzi et al., 2016) presented a p-value lower than 0.01 ($p < 0.01$).	8-13
Certainty of evidence	22	The total population across the nine clinical trials comprised 646 patients with T2DM: 325 in the experimental (probiotic) group and 321 in the control (placebo) group. Patient characteristics were comparable between groups, with ages ranging from 18 to 75 years. In all included trials, the following parameters were evaluated in both arms: fasting plasma glucose (FPG, mg/dL), HbA1c (%), and insulin concentration (μIU/mL). Among the selected studies, all assessed FPG, seven evaluated insulin levels, and six measured HbA1c.<br><br>The mean difference between the experimental and control groups (common effect model: MD = -17.67; 95% CI: -21.72 to -13.63; $p < 0.0001$; $I^2 = 0\%$; p for heterogeneity = 0.76; Figure 23) indicates a significant reduction in fasting plasma glucose (FPG) levels in the experimental group following probiotic supplementation. No heterogeneity was observed among the studies, as reflected by the I^2 value of 0.0% (95% CI: 0.0% to 64.8%). Additionally, a funnel plot analysis of these nine studies (Figure 3 - Left) revealed an overall symmetrical	8-13

Section and Topic	Item #	Checklist item	Location where item is reported
		distribution, suggesting no significant publication bias (Figure 3 - Right). However, several studies exhibited high standard errors, such as those by Tajabadi-Ebrahimi et al. (2016), Khalili et al. (2018), Jiang et al. (2024), and Savytska et al. (2024).28,29,30,31 We generated a contour-enhanced funnel plot (Figure 3 - Left), which helped clarify the statistical significance. The studies with a significant effect size ($p < 0.01$) were identified as three: Asemi et al. (2013), Zikou et al. (2023), and Firouzi et al. (2016).<br><br>At the end of the treatment, the probiotic groups showed a significantly greater reduction in mean difference compared to the placebo group (common effect model: MD = -0.42; 95% CI: -0.67 to -0.17; $p = 0.0009$; $I^2 = 0.5\%$; p for heterogeneity = 0.40; Figure 4). This analysis included data from five studies, as not all nine trials reported this parameter. The funnel plot (Figure 5 - Left) demonstrated an overall symmetrical distribution. A very low degree of heterogeneity was observed ($I^2 = 0.5\%$; 95% CI: 0.0% to 79.3%).30,31,32,33,35<br><br>However, the data also exhibited an asymmetrical pattern, which prompted the generation of a contour-enhanced funnel plot (Figure 5 - Right). This analysis identified that the studies showing a significant effect ($p < 0.01$) were those by Madempudi et al. (2019) and Jiang et al. (2021).<br><br>The mean difference indicates a significant effect between groups at the end of the study (common effect model: MD = -2.26; 95% CI: -2.87 to -1.64; $p < 0.0001$; $I^2 = 0\%$; p for heterogeneity = 0.67; Figure 6). This analysis included data from six studies. The funnel plot (Figure 7 - Left) shows that all studies fall within the expected funnel shape, suggesting the absence of publication bias. A low degree of heterogeneity was observed ($I^2 = 0.0\%$; 95% CI: 0.0% to 74.6%). We also generated a contour-enhanced funnel plot (Figure 7 - Right), which revealed that only two studies (Asemi et al., 2015 and Firouzi et al., 2016) presented a p-value lower than 0.01 ($p < 0.01$).	
**DISCUSSION**			
Discussion	23a	Diabetes mellitus is a well-known condition involving several organ systems. It can complicate the natural history of other diseases (e.g., cardiovascular, neurological, oral, etc.), having a two-way interaction with those.37 In recent years, several trials have been conducted to evaluate the role of probiotics and nutraceuticals in supporting insulin therapy.38,39,40<br><br>The present meta-analysis aimed to evaluate the efficacy of probiotics in patients receiving treatment for T2DM. Among the probiotics most frequently used as adjuncts to pharmacological therapy for T2DM in the scientific literature are Lacticaseibacillus rhamnosus, Limosilactobacillus reuteri, Akkermansia muciniphila, and members of the Bifidobacteriaceae family.21<br><br>In the selected clinical trials, the most frequently represented probiotics were Lactobacillus acidophilus and Lacticaseibacillus casei (7/9), Bifidobacterium bifidum and Lactobacillus delbrueckii subsp. lactis (4/9), Bifidobacterium longum (3/9), L. reuteri (2/9), and Lacticaseibacillus rhamnosus (1/9) This study evaluated three parameters—FPG, HbA1c, and insulin concentration—and conducted three separate meta-analyses. At the conclusion of the analysis, only three studies showed a significant decrease in FPG, three studies in HbA1c, and two studies in insulin concentration. Probiotic administration exerted a positive effect on patients with T2DM, as clinical improvements were reported by the end of the studies, or the measured parameters remained stable.	14
	23b	This study evaluated three parameters—FPG, HbA1c, and insulin concentration—and conducted three separate meta-analyses. At the conclusion of the analysis, only three studies showed a significant decrease in FPG, three studies in HbA1c, and two studies in insulin concentration. Probiotic administration exerted a positive effect on patients with T2DM, as clinical improvements were reported by the end of the studies, or the measured parameters remained stable.<br><br>Our meta-analysis, based on the reviewed clinical trials, demonstrates the efficacy of specific probiotics in supporting the treatment of T2DM. Although the findings are derived from only nine clinical trials, each with relatively small sample sizes and heterogeneous patient characteristics (age and gender), this study represents a meaningful advancement in diabetes therapy. Nevertheless, further research is required to confirm the long-term safety and efficacy of probiotic supplementation.	14-15

Section and Topic	Item #	Checklist item	Location where item is reported
	23c	This study evaluated three parameters—FPG, HbA1c, and insulin concentration—and conducted three separate meta-analyses. At the conclusion of the analysis, only three studies showed a significant decrease in FPG, three studies in HbA1c, and two studies in insulin concentration. Probiotic administration exerted a positive effect on patients with T2DM, as clinical improvements were reported by the end of the studies, or the measured parameters remained stable.<br>Our meta-analysis, based on the reviewed clinical trials, demonstrates the efficacy of specific probiotics in supporting the treatment of T2DM. Although the findings are derived from only nine clinical trials, each with relatively small sample sizes and heterogeneous patient characteristics (age and gender), this study represents a meaningful advancement in diabetes therapy. Nevertheless, further research is required to confirm the long-term safety and efficacy of probiotic supplementation.	14-15
	23d	This study evaluated three parameters—FPG, HbA1c, and insulin concentration—and conducted three separate meta-analyses. At the conclusion of the analysis, only three studies showed a significant decrease in FPG, three studies in HbA1c, and two studies in insulin concentration. Probiotic administration exerted a positive effect on patients with T2DM, as clinical improvements were reported by the end of the studies, or the measured parameters remained stable.<br>Our meta-analysis, based on the reviewed clinical trials, demonstrates the efficacy of specific probiotics in supporting the treatment of T2DM. Although the findings are derived from only nine clinical trials, each with relatively small sample sizes and heterogeneous patient characteristics (age and gender), this study represents a meaningful advancement in diabetes therapy. Nevertheless, further research is required to confirm the long-term safety and efficacy of probiotic supplementation.	14-15
**OTHER INFORMATION**			
Registration and protocol	24a	This meta-analysis is not registered.	
	24b	The protocol is not prepared.	
	24c	N/A	
Support	25	This study did not receive public funding or private grants.	15
Competing interests	26	Marica Colella is a Section Editor of the journal EMIDDT. All other authors disclose no conflict of interest.	15
Availability of data, code and other materials	27	N/A	

From: Page MJ, McKenzie JE, Bossuyt PM, Boutron I, Hoffmann TC, Mulrow CD, et al. The PRISMA 2020 statement: an updated guideline for reporting systematic reviews. BMJ 2021;372:n71. doi: 10.1136/bmj.n71

For more information, visit: http://www.prisma-statement.org/
